# Cognitive Overload: How to Protect Older Adults From Diminished Financial Decision-Making Capacity

**DOI:** 10.1093/geroni/igab046.2833

**Published:** 2021-12-17

**Authors:** Christopher Heye, Elizabeth Loewy, Katie Wade

**Affiliations:** 1 Whealthcare Planning, Belmont, Massachusetts, United States; 2 EverSafe, New yORK, New York, United States; 3 Whealthcare Planning, Belmont, Connecticut, United States

## Abstract

The aging population in the US poses a major threat to the financial security of older adults and their families. Millions of older adults will need to successfully navigate a multitude of financial and legal issues if they are to safely manage their assets while they are alive, and then securely transfer trillions of dollars to their heirs in accordance with their wishes. But most older adults are less healthy than their younger counterparts, and 25% or more over 65 are likely to suffer from diminished decision-making capacity. In short, older adults in the US will have to make some of the most important financial decisions of their lives just as their decision-making capacity is in decline. We offer recommendations to make it easier for financial services firms, medical professionals, non-profit organizations, and technology companies to work together to find better solutions for managing the complex issues around diminished decision-making capacity that is only likely to worsen in the years ahead.

